# A clinical review of phototherapy for psoriasis

**DOI:** 10.1007/s10103-017-2360-1

**Published:** 2017-10-24

**Authors:** Ping Zhang, Mei X. Wu

**Affiliations:** 1 Department of Dermatology, Wuhan No.1 Hospital, Tongji Medical College, Huazhong University of Science and Technology, Wuhan, Hubei 430022 China; 2 Wellman Center for Photomedicine, Massachusetts General Hospital, and Department of Dermatology, Harvard Medical School, Boston, 02114 MA USA

**Keywords:** Phototherapy, Psoriasis, Laser, Ultraviolet, Low-level light/laser

## Abstract

Psoriasis is an autoimmune inflammatory skin disease. In the past several decades, phototherapy has been widely used to treat stable psoriatic lesions, including trunk, scalp, arms and legs, and partial nail psoriasis. A variety of light/lasers with different mechanisms of action have been developed for psoriasis including ultraviolet B (UVB), psoralen ultraviolet A (PUVA), pulsed dye laser (PDL), photodynamic therapy (PDT), intense pulsed light (IPL), light-emitting diodes (LED), and so on. Because light/laser each has specific therapeutic and adverse effects, it is important to adequately choose the sources and parameters in management of psoriasis with different pathogenic sites, severities, and duration of the disorder. This review aims at providing most updated clinic information to physicians about how to select light/laser sources and individual therapeutic regimens. To date, UV light is primarily for stable plaque psoriasis and PDL for topical psoriatic lesions with small area, both of which are safe and effective. On the other hand, PUVA has better curative effects than UVB for managing refractory psoriasis plaques, if its side effects can be better controlled. PDL provides optimal outcomes on nail psoriasis compared with other lasers. Although the trails of low-level light/laser therapy (LLLT) are still small, the near infrared (NIR) and visible red light with low energy show promise for treating psoriasis due to its strong penetration and encouraging photobiomodulation. IPL is rarely reported for psoriasis treatment, but PDT-IPL has been found to offer a moderate effect on nail psoriasis. In brief, various phototherapies have been used either in different combinations or as monotherapy. The modality has become a mainstay in the treatment of mild-to-moderate psoriasis without systemic adverse events in today’s clinical practice.

## Introduction

Psoriasis is a chronic, recurrent, and immune-mediated inflammatory disease that affects 2–3% of the world population. It is associated with genetic predisposition, autoimmune disorders, psychiatry and psychological health, environmental factors (e.g., infection, stress, trauma), and so on. The pathogenesis is closely related to abnormal interactions among innate immunity, T cells, keratinocytes, etc. Immune cells in the patients release excess proinflammatory factors, leading to uncontrollable activation of congenital and acquired immune system, such as nuclear factor-κB (NF-kB) signaling pathway and differentiation of T helper (Th) cells toward Th1 and/or Th17 cells [1]. The complex pathogenesis results in tissue and organ damage over time, manifested by hyperproliferation, inflammation, and other clinical syndromes at the lesion sites. Therapeutic options for psoriasis can be divided into two aspects: systemic and topical treatments. The former involves immune inhibitors, like methotrexate, cyclosporine; retinoids (acitretin); immune modulators, such as glycyrrhizin, leflunomide [2]. Additionally, newly developed biological agents have been employed to treat moderate to severe psoriasis with body surface area (BSA) greater than 10% or psoriasis area and severity index (PASI) higher than 10 [3], including tumor necrosis factor α antagonists (etanercept, infliximab, etc.), alefacept, efalizumab, and ustekinumab [4]. As for topical treatment that is mainly for mild or moderate psoriasis, it includes ointments (e.g., calcipotriol, calcineurin inhibitors, tretinoin, glucocorticoid), medicated bath with diastase or herbal extracts, and phototherapy. Phototherapy is an effective, safe, and accessible treatment without incurring any systemic side effects, in contrast to biologic agents or other drugs, especially for stable plaque psoriasis. Moreover, phototherapy can be combined with biologic agents for the treatment of severe psoriasis [5].

Although phototherapy is convenient to use without severe adverse events, inadequate choice of laser/light types or parameters or unnecessary laser exposure could cause erythema, skin burning, photoaging, etc. It is therefore critical for clinicians to properly choose a right light source for a special type of psoriasis. We review the current literatures and focus on recent developments in psoriasis phototherapy by comparing curative effects between commonly used therapies and some new methodologies. We also update information with respect to their mechanisms of action in an attempt to provide some clinical guidance for psoriasis phototherapy.

## Phototherapy for psoriasis

Many types of phototherapy have been developed and used for the treatment of psoriasis over the last few decades as summarized in Table 1. Among them, broadband ultraviolet B light (BB-UVB, 290-320 nm) was first developed, but was later replaced by narrowband ultraviolet B (NB-UVB, 311 nm) as the latter is more effective than the former. The excimer laser/lamp of 308 nm was next invented in 1997 and used as a monochromatic UVB source for psoriasis treatment. The advantage of using excimer is its targeting ability that can spare unaffected skin while providing high doses to the skin involved. Studies found that the 308-nm excimer lamp is as effective in clearance of psoriasis as the excimer laser [6]. Both NB-UVB and excimer laser are currently used as the first-line therapy for stable plaque psoriasis. A conventional photochemotherapy using UV is called psoralen ultraviolet A (PUVA), which combines a photosensitizing drug and ultraviolet radiation. PUVA can be either systemic (oral, injection) or bath/cream-PUVA, both of which have been used to treat plaque psoriasis in stationary phase [7]. Some researchers selected PUVB to treat plaque psoriasis and found that PUVB had the similar curative and side effects as PUVA [8]. Apart from UV light, the flash lamp pumped pulsed dye laser (PDL) has a wavelength of 585–595 nm, which targets the chromophore hemoglobin and can selectively damage vessels, so PDL is the preferred laser for congenital and acquired vascular lesions [9]. PDL was first used to treat psoriasis in 1992 by Hacker and Rasmussen [10]. PUVA and PDL become the second-line therapy for plaque psoriasis, with preference of PUVA to refractory psoriatic plaques and PDL to nail psoriasis (Table 1). Finally, low-level light/laser therapy (LLLT) has been widely applied in dermatology. It has been recently considered to be effective for psoriasis treatment. A preliminary study investigated efficacy of combination of 830 nm (near infrared) and 630 nm (visible red light) emitted by light emitting diode (LED) to treat recalcitrant psoriasis [11]. Because of its ability of stronger penetration and potential photobiomodulation, LLLT has a promising expectation. In the future, other types of laser and light sources should be explored for the treatment of psoriasis.

**Table 1:** Summary of phototherapeutics for psoriasis

Classification of light source	Sub-light source	Wavelengths	Indications
First-line therapy			
UVB	NB-UVB	311 nm	Stable plaque psoriasis, > 10% body surface
	excimer laser/lamp	308 nm	Topical plaque psoriasis, non-pustular palmoplantar psoriasis
Second-line therapy			
PUVA	bath/cream-PUVA	320–400 nm	Refractory psoriatic plaques, palmoplantar pustular psoriasis
	Oral-PUVA	320–400 nm	Stable plaque psoriasis, palmoplantar psoriasis
PDL		585–595 nm	Nail psoriasis
Third-line therapy			
PDL		585–595 nm	Topical plaque psoriasis
PDT	LED		chronic plaque psoriasis, Nail psoriasis
	He-Ne	632.8 nm	
	IPL	555–950 nm	
Red light		620–770 nm	Plaque psoriasis
Blue light		400–480 nm	Plaque psoriasis
NIR		830 nm, 810 nm	Plaque psoriasis
Excimer		308 nm	Nail psoriasis
IPL		550–950 nm	Plaque psoriasis
PUVB		290–320 nm	Stable plaque psoriasis
BB-UVB		290–320 nm	Stable plaque psoriasis
Sunbath		400–760 nm	Chronic plaque psoriasis

## Clinical effects of UV light and the regulatory pathways

UV phototherapy is a popular treatment for psoriasis. Specifically, UVB therapy is generally for lesions covering at least 10% of body surface. In some specific cases, e.g., palmoplantar psoriasis that covers much less body surface can be also treated with UV light. In comparison with BB-UVB, NB-UVB clears psoriatic lesions faster and produces longer remissions with a good safety profile. Typical regimens of NB-UVB involve dosing three times per week for at least 3 months [12], and an appropriate initiating dose is usually 50% of the minimal erythema dose (MED) and/or simultaneously determined according to Fitzpatrick skin type. A randomized double-blind comparison of high dose and low dose of NB-UVB radiations in psoriasis patients has revealed that both regimens have comparable clearance rates, but the high-dose radiation prolongs remission with a fewer sessions of treatment [13]. Moreover, NB-UVB can be combined with other topical therapies, such as emollients, calcipotriene, and coal tar, to significantly improve its efficacy [14, 15]. It can also be combined with systemic agents in moderate-to-severe psoriasis patients, such as methotrexate and mycophenolate mofetil [16]. Acitretin is a retinoid; it can regulate the differentiation and proliferation of epidermal cells, and it can be used in the treatment of severe psoriasis alone or safely combined with phototherapy. A combination of oral retinoids and NB-UVB reduced treatment time, recovery frequency, and dose-limiting side effects [6]. Excimer laser/lamp with limited spot size, which emits a light of 308 nm via xenon-chloride gases, is more applicable to psoriasis lesions at a size of less than 10% body surface, for instance, in palms, soles, elbows, or knees. The excimer laser was found to be as efficacious as topical PUVA in non-pustular palmoplantar psoriasis [17].

Psoriasis responds to UV radiation favorably, but the underlying mechanisms are not well understood. It is known that UVB induces apoptosis of pathogenic T cells and keratinocytes, inducing local and systemic immunosuppression. Accumulating evidence suggested that Th17 and T regulatory (Treg) cells play critical roles in the pathogenesis of psoriasis. Th17 pathway was downregulated by NB-UVB in psoriatic epidermis [18], as suggested by significant decreases of IL-17A, TNF-α, and IL-6 mRNA in peripheral blood mononuclear cells (PBMCs) in psoriasis patients following NB-UVB treatment [19]. In addition, IL-17F polymorphism might also affect the response to NB-UVB phototherapy [20]. Strikingly, downregulation of Th17 pathway was not accompanied with enhanced Th1 responses. Instead, UV treatment triggered ubiquitination and downregulation of the type I interferon (IFN) receptor chain IFNAR1 and thereby suppressed IFN signaling and the expression of related cytokines [21]. In this regard, Treg dysfunction and down-regulation are associated with psoriasis. Therefore, NB-UVB and PUVA increase Treg cells and restore Treg function via the upregulation of FOXP3 in Treg cells, alleviating psoriasis [22]. Another possibility is that reduced plasmin (a potent inflammatory cell activator) levels may be ascribed to the cumulative dose of NB-UVB, which also contributed to the therapeutic effect of NB-UVB on psoriasis [23].

PUVA is the gold standard of photochemotherapy modalities mainly for topical psoriatic plaques. Psoralen acts by intercalation of cellular DNA and forms monoadducts that induce apoptosis upon exposure to UVA. PUVA-induced apoptosis in HaCaT keratinocytes was thought to be induced by *Homo sapiens* microRNA-4516-mediated downregulation of STAT3/CDK6/UBE2N [24]. Studies have shown a better efficacy of PUVA, compared to UVB therapy on palmoplantar pustular psoriasis which is characterized by repeated eruptions of sterile pustules on the palms and soles, but it is without any differences between bath and oral-PUVA [25]. However, its application is limited owing to topical stimulation reaction, weak percutaneous permeability, gastrointestinal symptoms associated with systemic psoralens, and concerns over their long-term carcinogenic risk [26]. The efficacy and safety of topical PUVA was improved by liposomal nanocarriers of psoralen in imiquimod-induced psoriatic plaque model because the nanoparticle-packaged psoralen enhanced skin penetration of the drug and reduced its adverse effects on burning, blister, and pigmentation [27]. However, its clinical benefits remain to be determined.

Both PUVA and NB-UVB are effective therapies in psoriasis. However, considering the potential risks, dermatologists prefer NB-UVB as the first-line phototherapy (Table 1). If the systemic inflammation is under control in psoriasis pustular or erythrodermic psoriasis, NB-UVB can be also used to shorten the treatment time. If NB-UVB for the treatment of refractory plaque psoriasis in adults is invalid or does not make any progress, PUVA either alone or in combination with topical medications can be an alternative. UVB and PUVA can also be employed in pediatric psoriasis if children are mentally and psychologically able to tolerate the procedure and accept eye protection. Bath and cream PUVA are preferred over oral PUVA in pediatric psoriasis, but should be restricted to those aged over 10 or 16 years, respectively [28].

## Pulsed dye laser treatment and its mechanism

PDL is the preferred therapy for most superficial cutaneous vascular lesions. In recent years, it has been used in some clinical researches to treat non-vascular indications, such as psoriasis, acne vulgaris, papulopustular rosacea, hypertrophic scar, and so on [29]. Erceg et al. [30] have reviewed the efficacy of PDL for inflammatory skin disease and concluded that PDL is an effective and safe method to treat localized plaque psoriasis. In accordance with this, a prospective randomized controlled study was performed to compare the efficacy of PDL with UVB-TL01 in plaque psoriasis. PDL parameters used were 585 nm, a pulse duration of 0.45 ms, and a spot size of 7 mm with spot overlapping about 20% at fluencies 5.5–6.5 J/cm^2^ for a total of four treatments at an interval of 3 weeks between two treatments. Improvement of psoriasis lesions was noted at the 13th week, yet with no significant differences between PDL and UVB [31]. A within-patient controlled prospective trial of treatment of localized plaque psoriasis with excimer (twice weekly) and PDL (every 4 weeks) showed that excimer and PDL were useful treatments for plaque psoriasis with long-term remission, but psoriasis severity index (PSI) improvement was significantly greater in excimer than PDL [32]. Studies of PDL in psoriasis also reported low clearance rate, which could be improved by applying keratolytics with salicylic acid on psoriatic plaques before PDL treatment [33].

In spite of its effectiveness in clearing psoriasis plaques, the mechanism underlying PDL therapy has not yet been elucidated. It has been shown that the levels of VEGFR-2, 3, and E-selectin protein were reduced in lesions after PDL treatment, followed by downregulation of TNF-α and IL-23p19 [34]. It was speculated that PDL mainly acted on the capillaries in the upper dermis. PDL-induced selective photothermolysis of hemoglobin in vessels was one of the mechanisms of the clearance of psoriatic lesions. Apart from photothermolysis, PDL also reduced activated and memory effector Th cells in the dermis and cytotoxic T cells in the epidermis in plaque psoriasis [35], concurrent with induction of cytokine expression and collagen formation [36]. Studies about PDL treatment of hypertrophic scar have shown that PDL is strongly absorbed by hemoglobin, leading to coagulation necrosis in vessels, hypoperfusion, and local hypoxia [37]. Moreover, PDL could also play a role in limiting the production of transforming growth factor and platelet-derived growth factor; it can stimulate matrix metalloproteinases and IL-6 for matrix degradation [38, 39]. However, how these gene transcriptional activities are triggered by PDL remains largely unknown.

## Low-level light/laser therapy for psoriasis and the mechanism of action

In recent years, LLLT has been widely applied in dermatology. LLL is also called “cold laser,” which involves ultraviolet, visible, and near infrared with much lower energy densities than those lasers used for ablation, cutting, and thermally coagulating tissues. Light sources of LLLT include LED, helium-neon (He-Ne, 632.8 nm), and gallium arsenide (GaAs) laser. LED is the complex semiconductor that converts electrical current into incoherent narrow spectrum light. LED or lasers at a wavelength ranging from 600 to 1070 nm have been widely applied to LLLT. Although longer wavelengths penetrate deeper, lasers at 700-770 nm have been found to limit biochemical activity [40, 41]. Blue light (400-480 nm) can reduce the proliferative activity of keratinocytes, modulate T cell immune responses, and safely improve plaque psoriasis [42]. It is thus propitious to treat chronic hyperplastic and inflammatory dermatosis, such as psoriasis and atopic dermatitis. A prospective randomized study by comparing the efficacy of blue light (420 and 453 nm, LED) in treatment of psoriasis vulgaris once daily for 4 weeks showed significant improvement in either wavelength [43]. Red light (620–770 nm) is able to deeply penetrate skin to about 6 mm [44], stimulate mitochondrial activity, and modulate cytokine release from macrophages to reduce topical inflammation [45]. When patients with plaque psoriasis were treated sequentially with LED delivering continuous 830 and 633 nm in two 20-min sessions for 4 or 5 weeks, 60–100% of clearance rates were achieved without any significant side effects [11].

LLL is characterized by its ability to induce photobiological processes in cells. For instance, laser at 810 nm was shown to activate NF-kB in primary murine embryonic fibroblasts cells [46]. In contrast, a combination of curcumin (anti-proliferation) with LED blue light, along with red light radiation in psoriasis, inhibited NF-κB activity, activated caspase-8/9, and downregulated the phosphorylation level of Akt and ERK [47]. A growing number of investigations have demonstrated that LLL can improve mitochondrial function under stress by increasing ATP synthesis, reactive oxygen species (ROS) generation, and cell redox activity [40, 48]. The beneficial functions of LLLT rely on its ability to activate cytochrome c oxidase at the mitochondrial respiratory chain. Cytochrome c oxidase has strong absorption of light at wavelengths ranging from 670 to 830 nm [49]. Recent studies also showed that LLLT increased mitochondrial biogenesis in megakaryocytes, facilitating platelet biogenesis both in vivo and in vitro in preclinical studies [50]. Finally, laser irradiation at green, red, or infrared wavelength with special parameters can change gene expression and release of various mediators in human and animal cells. [51]. LLLT has been also commonly used in a variety of conditions for acceleration of healing and relief of pain and inflammation, etc. [52, 53]. Its advantages of non-invasion, few side effects, and measurable benefits merit to be explored in the treatment of psoriasis.

## Others

Photodynamic therapy (PDT) using 5-aminolevulinic acid (ALA) as photosensitizer is effective for superficial skin tumors partially because of its selective cytotoxic effect. The light sources of PDT include LED, He-Ne, IPL, and other visible lights. In recent years, PDT was employed to treat some inflammatory dermatoses (e.g., severe acne, rosacea, and psoriasis). PDT stimulated diverse immune cells to produce various cytokines, such as IL-1β, IL-2, and TNF-α. And fibroblasts secreted MMP-1 and 3 in response to PDT [54, 55]. The benefit of PDT to inflammatory dermatoses is probably ascribed to its suppression of TGF-β1 expression, while increasing IL-10 production, which was demonstrated in cultured fibroblasts after ALA-IPL-PDT [56]. However, the study of PDT-methyl aminolevulinate suggested a low proportion of psoriasis patients with complete remission, only six out of 17 [57]. Topical ALA-PDT was inadequate for chronic plaque psoriasis because of variability in clinical responses and severe pain. Single IPL has been applied to vascular skin disease, such as facial telangiectasias and port-wine stains. At present, IPL has been widely used to photorejuvenation as non-invasive therapy either alone or in combination with the photosensitizer [58]. A fewer literatures reported that IPL with 550-nm filter was effective to plaque psoriasis [59]. In this regard, sunlight contains mainly UV and visible and infrared light, and UVB from the sun works the same way as UVB in phototherapy. Thus, multiple and short exposures to sunlight are recommended in psoriasis patients if they are tolerant to sunlight in the basis of clinical studies of phototherapy. Yet, natural sunbath is so unbound, rendering patients to the risks of photosensitivity and sunburn increase, and thus it should be carefully monitored. The patients should apply broad-spectrum sunscreen to all areas of unaffected skin and wear sunglasses during sunbath.

## Phototherapy for nail psoriasis

Up to 50% of psoriasis patients have nail changes. Patients with psoriatic arthritis have an 80 to 90% lifetime incidence of developing nail psoriasis [60]. The manifestations of nail psoriasis include nail plate pitting, onycholysis, nail bed discoloration, sub-ungual hyperkeratosis, etc. Psoriatic nails tend to be progressive if left untreated and can significantly impair patient’s quality of life. The management of nail psoriasis includes patient education, topical ointments, and systemic therapy (e.g., methotrexate, acitretin, cyclosporine A, biologic agents). Phototherapy is useful when few nails are involved. In 2009, PDL was firstly used to treat nail psoriasis in which 61 nails were treated with PDT (MAL + PDL) and 60 nails treated with PDL alone. The nails treated with PDL had 33% improvement after 3 months and 58% improvement after 6 months in the basis of the nail psoriasis severity index (NAPSI) score, but it was without no statistical differences between PDT and PDL [61]. Other studies investigated PDL with different pulse durations and energy densities were also effective and well-tolerated to treat nail psoriasis [62]. A single-blinded comparison study of excimer laser and PDL, in which patients’ right hand nails were treated with excimer laser for twice weekly and the left hand nails with PDL for once every 4 weeks, showed that NAPSI improvement was greater in PDL than excimer group after 12 weeks [63]. A followed-up study in which finger and toe nails of 20 patients with psoriasis were treated by IPL at 550 nm exhibited improvement in the nail bed (71.2%) and nail matrix (32.2%) after a mean of 8.63 ± 3.6 treatment sessions [59]. However, the penetration of UVA and UVB was extremely poor in the human fingernails and the nail plates completely blocked UVB. The mean penetration of UVA was 1.65% and thus therapeutic effect of UV for nail psoriasis might be poor ^[^ 64 ^]^.

## Potential risks

The short-term side effects of phototherapy are generally mild and self-limiting, such as erythema, edema, pruritus, pain, purpura, transient petechiae, blister, and crusting, which occur during treatment or within the first 24 h after the treatment. The main long-term side effects include pigmentary disorder, photoaging, cataracts, and carcinogenesis. Photodermatitis usually occurs in UV radiation as UV-induced DNA damage or mutations result in activation of oncogenes or silencing of tumor-suppressor genes, which are closely related to the pathogenesis of skin squamous-cell carcinoma [65]. Furthermore, UV radiation was associated with melanoma. The children younger than 12 years of age who sustained frequent episodes of sunburn or UV exposure had a 3–4-fold increased risk of melanoma [66]. To reduce the complications and side effects, physicians should undergo standard training and exclude the patients who are intolerant to light and display obvious phototoxic or photosensitive reaction under radiation and who are using drugs (e.g., sulfonamides, fluoroquinolones, coal tar, tazarotene) that can impose a risk of photosensitivity. Patients using these drugs should talk with a doctor before treatment. In addition, pregnant women and those who are suffering from cardiac/cerebrovascular diseases, preexisting or manifest skin malignancy cannot receive phototherapy.

## Summation and future directions

Psoriasis is an inflammatory skin disease, involving the complex interaction network among which a variety of cells respond to light radiation differently. The outcome of phototherapy depends on a delicate balance between beneficial and detrimental effects of a specific laser. In comparison with other laser modalities, PUVA and UVB have the advantages of large radiation sizes, low price, and efficacy and safety that have been intensively demonstrated. In addition, PUVA has better effects than UVB on refractory psoriasis plaque and palmoplantar pustular psoriasis, but its side effects limit its broad application (Table 1). PDL provides optimal outcomes on nail psoriasis compared with other lasers. The trails of LLLT are still limited, but the NIR and visible red light with low energy show prospects for treating psoriasis due to its strong penetration and encouraging photomodulation. IPL is rarely reported for the treatment of psoriasis, but PDT-IPL has been found to offer a moderate effect on nail psoriasis.

Light spectra have different depths of penetration in skin, which can be applied to different target cells or tissues to obtain certain effects. In comparison with sunlight, if the curative effects of a light source, especially non-monochromatic light, are not as effective as sunlight or comparable, it does not have high clinical values. Apparently, artificial light has the advantage that can deliver high-dose radiation to the target in a short time, which may be also one of the therapeutic mechanisms. It is thus possible that combination of various monochromatic lights acting on different targets can be a ground-breaking way to improve outcomes of phototherapy in the future.
